# Patent Foramen Ovale Occlusion in Elderly Patients: Is It Worth It? A Large, Single-Center Retrospective Analysis

**DOI:** 10.3390/jcm13123514

**Published:** 2024-06-15

**Authors:** Sebastiano Gili, Giuseppe Calligaris, Giovanni Teruzzi, Giulia Santagostino Baldi, Manuela Muratori, Piero Montorsi, Daniela Trabattoni

**Affiliations:** 1 Centro Cardiologico Monzino, IRCCS, Via Parea, 4, 20138 Milan, Italy; 2 Dipartimento di Scienze Cliniche e di Comunità, Università degli Studi di Milano, 20122 Milan, Italy

**Keywords:** PFO closure, elderly, outcomes

## Abstract

**Background:** Patent foramen ovale (PFO) is often diagnosed in patients with cryptogenic stroke, aged > 60–65 years, but few data report the outcomes of PFO closure in elderly patients. **Methods:** Consecutive patients undergoing PFO closure at a single institution between January 2006 and December 2011 were included. Baseline clinical features and cerebral imaging data were collected, and a RoPE score was calculated for each patient. Procedural data were recorded as well as medical therapy upon discharge. All-cause death, ischemic stroke, TIA and systemic embolism recurrence at long-term follow-up were investigated, as well as new atrial fibrillation onset. **Results**: Overall, 462 patients were included, of whom 64 (13.8%) were aged ≥ 65 years. Female gender was slightly more prevalent in the younger group while hypertension was more frequent among elderly patients. Previous stroke/TIA was the indication for PFO closure in 95.3% of older patients and 80.4% of younger patients, whereas other indications were more frequent among younger patients. RoPE scores were lower in older patients (median RoPE score of 5 vs. 7), and atrial septal aneurysm was more frequently detected among elderly patients. All procedures were technically successful. Procedural or in-hospital complications equally occurred in 5 (7.8%) older patients (4 AF and 1 device embolization) and 30 (7.5%) young patients (29 AF or other supraventricular arrhythmias and 1 device embolization). The follow-up duration was longer among younger patients. All-cause mortality was higher in older patients (16 deaths vs. 4 at follow-up, log-rank *p* < 0.001), no recurrent strokes occurred, and 2 TIAs were reported among non-elderly patients. New-onset atrial fibrillation occurred in three elderly and eight young patients. **Conclusions**: PFO closure is a safe procedure in patients aged ≥ 65 years, associated with favorable long-term follow-up and the prevention of ischemic neurologic recurrences.

## 1. Introduction

Patent foramen ovale (PFO) closure has established itself as a safe and effective procedure to treat selected patients suffering from cryptogenic stroke [1,2,3,4,5,6]. Available guidelines for the management of patients with PFO, however, lack definite recommendations for older (≥60–65 years) patients with a PFO-related cerebrovascular event [7,8,9,10,11,12], a complex and large group of patients who were mostly excluded from PFO closure clinical trials. Only the DEFENSE-PFO trial included patients older than 65 years [6]. Nevertheless, in routine clinical practice, we have to face decisions on the management of patients with PFO and cryptogenic stroke aged ≥ 65 years: several studies have shown a higher prevalence of PFO among older patients with cryptogenic stroke [13], and its presence has been associated with an increased risk of recurrent events [14]. Additionally, the sub-analysis from the DEFENSE-PFO trial [6] and observational studies have shown the safety and preliminary efficacy of PFO closure in older PFO-related stroke patients [15]. The PFO causes more paradoxical embolism in old and frail people due to their increased propensity to venous thromboembolism, representing the precondition for paradoxical embolization. In fact, the prevalence of atherosclerotic disease and venous thrombosis has a steeper growth with older age and comorbidities. The absolute risk for patients with PFO increases with age and disease. However, risks and benefits in long-term follow-up are not available so far and only anecdotal reports and small subgroup analyses have been published.

Among available guidelines and consensus documents, AHA/ASA (American Heart Association/American Stroke Academy) does not recommend PFO closure in subjects older than 60–65 years. The American Academy of Neurology suggests the possibility of PFO closure in patients between 60 and 65 years as a Level C indication, while only the SCAI (Society for Cardiovascular Angiography and Interventions) suggests PFO closure rather than long-term antiplatelet therapy alone. The European position paper on the management of patients with PFO emits a possible indication in patients over 65 years, despite the lack of evidence, taking into account the age-related confounder and the risks of interventional procedures, on a case-by-case basis. Finally, the expert panel recently releasing the European Stroke Organization (ESO) guidelines on the diagnosis and management of patent foramen ovale encourages the inclusion of patients older than 60 years old with stroke and PFO in randomized trials whenever possible, or at least in a registry, given the impossibility to provide any evidence-based recommendation [16]. The accurate patient selection needs, especially in older patients, include atrial fibrillation risk factor assessments as well as non-invasive imaging to detect subclinical atherosclerotic disease [17].

We designed the present study to assess the main features of elderly patients treated with PFO closure in a real-world cohort from a high-volume tertiary center and to evaluate the short- and long-term outcomes of these patients in comparison to younger subjects.

## 2. Methods

Consecutive patients undergoing patent foramen ovale occlusion at our institution between January 2006 and December 2011 were included. This is an observational, retrospective, monocentric and non-profit study. Indications to PFO occlusion were conducted according to clinical practice and consensus documents’ recommendations available at that time before randomized clinical trials demonstrating the superiority of PFO closure vs. standard therapy were definitely published. The indications were categorized as follows: 1. primary prevention, mainly based on high-risk PFO morphologic characteristics, including severe migraine not responsive to optimal medical treatment; 2. secondary prevention due to stroke, transient ischemic attack (TIA), systemic embolism or silent cerebral embolism detected at cerebral magnetic resonance (MR). Baseline clinical features were collected including biometric data and medical history. Cerebral imaging data (magnetic resonance or computed tomography, as available) were collected and, where necessary, reviewed, to assess the pattern of ischemic lesions (cortical ischemic lesions, sub-cortical small ischemic lesions or negative brain imaging). All examinations performed during the diagnostic workup leading to PFO closure were assessed, including carotid ultrasound, thrombophilic screening and ambulatory ECG monitoring (or loop recorder implantation), transcranial Doppler, transthoracic or transesophageal echocardiography. The RoPE scores were calculated in any patient [16]. In the case of the detection of atrial fibrillation, significant carotid atherosclerotic disease (stenosis ≥ 50%) or uncontrolled hypertension during diagnostic workup, patients were not referred for PFO closure.

Procedural data recorded included the type and size of the implanted device, the type of echocardiography guidance, procedural time, contrast dose and radiation dose. Acute residual shunt was searched in all patients with plain transthoracic echocardiography. Prescribed antithrombotic regimens upon discharge included single-antiplatelet therapy (SAPT) with a P2Y12 inhibitor, double-antiplatelet therapy (DAPT) with acetylsalicylic acid (ASA) and a P2Y12 inhibitor or anticoagulants associated with ASA or a P2Y12 inhibitor.

Procedures’ effectiveness and complications were recorded, as well as procedural data, including length and radiation exposure. Information on adverse events at follow-up was collected during remote (phone call) or on-site clinical visits, including all-cause death, recurrence of ischemic stroke, TIA or systemic embolism. For patients with migraine at baseline, symptom changes were documented. Data on new-onset atrial fibrillation or other arrhythmias, device-related complications, hemorrhagic events and the presence of residual shunt were collected as well. A total of 462 consecutive patients treated with PFO closure after cryptogenic stroke, between January 2006 and December 2011, were the subject of our analysis. The results obtained in patients older than 65 years have been compared with those in younger patients to evaluate the clinical outcomes, atrial fibrillation onset and recurrence of neurological events in the acute (in-hospital, intra- and post-procedural), mid-term (3 years) and long-term (>10 years) follow-up, and survival rate.

Inclusion and exclusion criteria of elderly patients

Inclusion criteria:

- Age ≥ 65 years; percutaneous PFO closure procedure performed during 2006–2011 at Centro Cardiologico Monzino; patient with at least one cryptogenic ischemic stroke event in the last 12 months (PFO closure performed in ischemic patients); patient’s signed informed consent form (ICF) or covenant to research (Patto alla ricerca di CCM) or telephone consent.

Exclusion criteria:

- Age < 18 years.
- Patient treated with PFO closure in primary prevention.
- Patient who expressly decided not to participate in Centro Cardiologico Monzino research.

Inclusion and exclusion criteria of younger patients

Inclusion criteria:

- Age ≥ 18 years and <65 years.
- Percutaneous PFO closure procedure performed in the period of 2006–2011 at Centro Cardiologico Monzino.
- Patient with at least one event of cryptogenic ischemic stroke in the last 12 months (PFO closure performed in ischemic patients).

### Sample Size Calculation and Statistical Analysis

With a sample size of 65 subjects (>65 years), a procedural effectiveness of 90% is estimated with a 95% confidence interval (normal approximation to the binomial calculation) of 83.7–97.8%.

A sample size of 470 subjects (65 aged >65 years and 405 aged <65 years) is needed to detect as significant (alpha = 0.05) a 10% reduction in MACE between the two groups, assuming an incidence in the reference group (<65 years) of 4% and a statistical power of 80%.

Continuous variables were reported as medians with interquartile ranges (IQRs) or means ± standard deviation (SD) and were compared using the Kruskal–Wallis non-parametric test; categorical variables are reported as numbers with percentages and were compared using Pearsons’s Chi-square test or Fisher’s exact test, as appropriate. Survival analyses and the incidence of AF at follow-up were evaluated with Kaplan–Meier curves and were compared using a log-rank test. A two-sided *p*-value of 0.05 was set as statistically significant. All analyses were performed using Jamovi version 1.6.21.0.

## 3. Results

Overall, 462 out of the 470 consecutive patients (*n* = 8 lost to follow-up) were included, of whom 64 (13.8%) were aged 65 years or more. Female gender was slightly more prevalent in the younger group, even if the difference was not statistically significant. Hypertension was more frequent among elderly patients, whereas no differences were observed regarding all the other cardiovascular risk factors (Table 1). No patient reported coronary artery disease or chronic kidney disease.

Previous stroke/TIA was the indication for PFO closure in 95.3% of elderly patients and in 80.4% of younger patients, whereas other indications were more frequent among younger patients, including primary prevention (4.7% vs. 7.5%, *p* = ns), systemic embolism (1.6% vs. 4.8%, *p* = ns) and silent cardioembolic lesions (3.1 vs. 14.8%, *p* < 0.001) on brain MRI.

**Figure 1:**
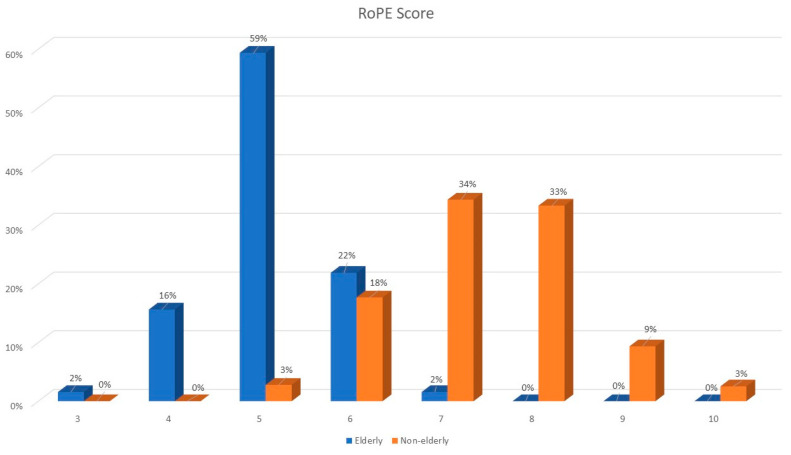
RoPE score distribution among elderly and younger patients.

RoPE scores were lower in older patients (median RoPE score of 5 vs. 7); see Figure 1. The right–left shunt detected using echocardiography after the Valsalva maneuver was similar between the two groups, whereas atrial septal aneurysm was more frequently observed among elderly patients.

### 3.1. Procedural Characteristics

The procedural data are summarized in Table 2. All procedures were performed under conscious sedation and intracardiac echo guidance with double disc-device implantations. No differences were observed in terms of the type of implanted device, with the vast majority of patients being treated with the Amplatzer PFO occluder (Abbott, Chicago, IL, USA) in both groups; non-elderly patients were more frequently treated with devices of different manufacturers. Dimensions of implanted devices were similar as well between the two groups, making the 25 mm device the most commonly used overall. Global procedural time was shorter among elderly patients, whereas no differences were observed regarding fluoroscopy time and radiation dose.

A very high technical success was observed equally between the patients’ groups. Procedural or in-hospital complications occurred in 5 (7.8%: *n* = 4 AF and *n* = 1 device embolization) elderly patients and in 30 (7.5%: *n* = 29 AF or other supraventricular arrhythmias, *n* = 1 device embolization) younger patients (*p* = ns).

### 3.2. Follow-Up

The antithrombotic therapy prescribed upon discharge did not differ among the two groups of patients, as almost the totality of the patients received double-antiplatelet therapy for at least 3 months, followed by single-antiplatelet treatment. Residual right-to-left shunt was detected in 9.3% of the patients, mostly of a mild grade, without severe residual shunt detected in the long-term follow-up (Table 2).

**Figure 2:**
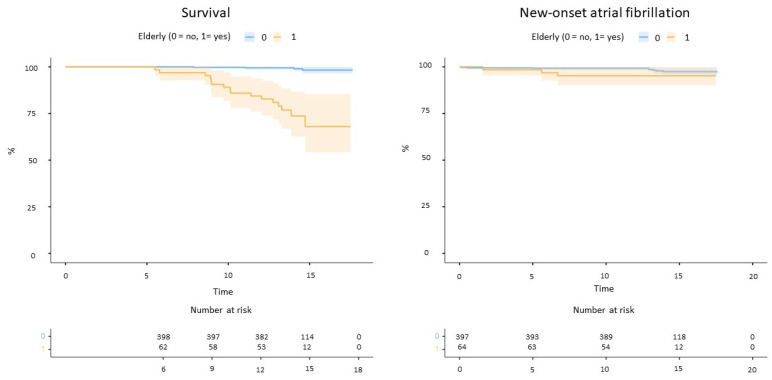
Mortality (**left**) and incident atrial fibrillation (**right**) in the study cohort.

The follow-up duration was longer for non-elderly patients (Table 1). Elderly patients reported significantly higher mortality for any cause compared to non-elderly (16 deaths vs. 4 at follow-up, log-rank *p* < 0.001, Figure 2). No strokes or systemic embolism occurred, whereas two TIAs were reported among non-elderly patients (one associated with device embolization). New-onset atrial fibrillation occurred in three elderly and eight non-elderly patients (log-rank *p* = 0.15, Figure 2).

## 4. Discussion

The present study shows that PFO closure in elderly patients is a common interventional procedure that requires a case-by-case tailored approach, given the scarcity of guidance offered by guidelines and consensus documents.

In our study, we demonstrated that PFO closure can be performed in older patients with good procedural results and without increased clinical risks or technical complications. Procedural data did not significantly differ between the study cohort, and in-hospital and short-term complications were rare among both groups. In long-term follow-up, beyond the higher any-cause mortality rate observed in older patients largely attributable to age itself, the rate of recurrent stroke was minimal for all patients, irrespective of age. Additionally, no differences in the incidence of induced atrial fibrillation after PFO closure were observed comparing older to younger patients, according to the atrial fibrillation incidence in the general population.

Despite usually being considered a relevant cause of cryptogenic stroke in younger patients, in whom it is often easier to rule out alternative causes of such events, PFO might also play a significant role in older populations. Actually, national and international PFO guidelines lack definite recommendations about transcatheter PFO closure for patients aged > 60 years. However, observational data have shown a higher prevalence of PFO among elderly subjects experiencing cryptogenic stroke, with an associated high risk of recurrent events.

Handke and colleagues [13] showed in a large prospective cohort that the prevalence of PFO was higher in older patients with cryptogenic stroke compared to patients with stroke of known origin. A subsequent meta-analysis similarly reported a PFO prevalence ranging from 16% to 38% among older patients with cryptogenic stroke compared to 8–23% among control patients, confirming the existence of a significant association between PFO and cryptogenic stroke also in this category of patients [14,15,16,17,18]. In the same study, it was estimated that the probability of PFO being an incidental finding was 20% among younger cryptogenic stroke patients and 48% in older patients, while high-risk anatomical characteristics (i.e., atrial septal aneurysm) reduced the PFO incidental finding down to 9% and 26%, respectively. Older patients (≥65 years) are also at higher risk of stroke/TIA recurrence following cryptogenic stroke in the presence of PFO [19]. It is also known that thromboembolic risks, especially venous thrombosis, increase with age [20]. Moreover, a large autoptic series has yielded two interesting findings regarding PFO in older patients, i.e., that the prevalence of PFO tends to lower during lifetime (from 34.3% during the first three decades to 20.2% in the ninth and tenth decades) and that the average dimension of PFO tends to increase with age [21]. Based on these observations, it is clear that the PFO closure is a procedure that has to be considered even in older, select patients.

Patient selection plays a crucial role in PFO closure, and this is particularly true for older patients. The RoPE score, which is validated and widely used [19], includes as main determinants patient’s age and the presence of risk factors for atherosclerosis, which are well known to be more prevalent in older patients. This is in fact reflective of the higher prevalence of competing causes of stroke in older patients, notably atrial fibrillation and atherosclerotic disease, which are caused mainly by those risk factors that are included in the RoPE score. For this reason, for older patients, the evaluation has to focus mainly on the anatomical feature of PFO associated with a higher risk of paradoxical embolism, such as atrial septal aneurism, shunt magnitude, long tunnel, prominent Eustachian Valve or Chiari’s network, and on the assessment of clinical and neuroradiological features of stroke, to determine the likelihood of its cardioembolic origin. Inherently, in our study, atrial septal aneurysm was more frequent in the older population, being reported in more than one-third of such patients.

Among the different randomized clinical trials assessing PFO closure, only the DEFENSE-PFO trial included patients aged ≥ 60–65 years. Despite being limited by the small sample size, the study drew an answer towards the effectiveness of PFO closure in older patients: in patients ≥ 60 years, the primary endpoint (ischemic stroke or transient ischemic attack (TIA) during 2 years of follow-up) was reached in 24.6% in the medically treated group and 0% in the closure group (HR, 7.36; 95% CI, 0.28 to 195.81; log-rank *p* = 0.07), whereas among patients aged ≥ 70 years, four out of six medically treated patients experienced the primary endpoint compared to none of the five patients treated with PFO closure. Further evidence comes from observational studies, which, similarly to our report, showed the overall good safety of PFO closure in older patients [22,23,24], with only one study reporting a higher rate of vascular complications in older patients [25]. A crucial factor in this setting remains, however, the occurrence of atrial fibrillation after PFO closure, as it has been reported as being more frequent in older patients [24]. In this setting, the value of implantable cardiac monitoring devices and smart watches remains to be explored: despite their known ability to detect silent atrial fibrillation prior to and following PFO closure [26], the role of atrial fibrillation occurring after PFO closure remains to be explored, as it has been demonstrated to be generally transient and self-resolving [4].

Definitely, limited evidence is available on the efficacy of PFO closure for elderly patients; a sub-analysis of DEFENSE-PFO with a median follow-up duration of 2.5 years showed a prominent efficacy of PFO closure for recurrent stroke in patients aged *>* 60 years after cryptogenic stroke [27]. On the other hand, a higher incidence of recurrent stroke due to aortic atheroma or atrial fibrillation and vascular carotid disease was observed in elderly patients in a mean 4.5-year follow-up [28]. The results of this study show a high rate of high-risk PFO (13.1%) in cryptogenic stroke and suggest that the presence of a high-risk PFO, even in patients over 60 years of age, contributes to the pathogenesis of cryptogenic stroke because other cardioaortic embolic pathologies were absent in these patients. Future studies are needed to clarify the effectiveness and safety of PFO closure in patients with cryptogenic stroke, regardless of age or degree of PFO.

## 5. Future Directions

Our results are another contribution to the mounting evidence suggesting that elderly patients should not be denied the opportunity to undergo PFO closure following cryptogenic stroke. This field has been overlooked until recently as PFO is commonly regarded as an issue only for younger patients. A recent analysis of data from major observational registries and all six randomized clinical trials on PFO closure has shown that applying strict selection criteria, including only patients with high-risk PFO features, a randomized clinical trial in elderly patients could be feasible [29].

## 6. Study Limitations

The study is retrospective and definite recommendations cannot be drawn from our results; thus, our current findings can be regarded as hypothesis-generating. Another limitation resides in the long timespan during which included patients were treated; even if during these years technical evolutions in PFO closure devices were limited, potential influences of changes in the approach to PFO treatment cannot be ruled out.

## 7. Conclusions

PFO closure can be performed safely in older patients with good clinical results during a short- and long-term follow-up. Patient selection is the main issue when assessing such patients and further studies are needed to improve the identification of patients benefiting from this procedure.

## Figures and Tables

**Table 1:** Baseline Clinical Characteristics.

	Overall	Elderly	Non-Elderly	*p*-Value
	(*n* = 462)	(*n* = 64)	(*n* = 398)	
**Age (years)**	**45 (37–54)**	**69 (66.8–71.0)**	**36 (43–49)**	**<0.001**
Male gender	194 (42.0)	32 (50.0)	162 (40.7)	0.17
Body mass index	23.5 (21.5–25.8)	24.2 (22.3–26.0)	23.5 (21.3–25.7)	0.24
**Hypertension**	**194 (42.0)**	**15 (57.7)**	**179 (41.1)**	**0.003**
Diabetes mellitus	4 (0.9)	3 (4.7)	1 (0.3)	0.102
Active smoke habit	20 (4.3)	5 (7.8)	15 (3.8)	0.25
Dyslipidemia	35 (7.6)	7 (10.9)	28 (7.0)	0.35
**RoPE score**	**7 (6–8)**	**5 (5–5)**	**7 (7–8)**	**<0.001**
History of DVT or PE	17 (3.7)	5 (7.8)	12 (3.0)	0.17
**History of stroke/TIA**	**381 (82.5)**	**61 (95.3)**	**320 (80.4)**	**<0.001**
Migraine	138 (29.9)	18 (28.1)	120 (30.2)	0.74
Aura	25 (5.4)	7 (10.9)	18 (4.5)	0.12
Oral contraceptives	28 (6.1)	0	28 (7.0)	
**PFO closure indication**				**<0.001**
Primary prevention	33 (7.1)	3 (4.7)	30 (7.5)	
Stroke	84 (18.2)	18 (28.1)	66 (16.6)	
TIA	297 (64.3)	43 (67.2)	254 (63.8)	
Systemic embolism	20 (4.3)	1 (1.6)	19 (4.8)	
Silent cardioembolic lesions	61 (13.2)	2 (3.1)	59 (14.8)	
Cerebral imaging findings				0.19
Cortical	117 (25.3)	25 (39.1)	92 (23.1)	
Subcortical	116 (25.1)	9 (14.1)	107 (26.9)	
Negative	229 (49.6)	30 (46.9)	199 (50.0)	
**Atrial septal aneurysm**	**109 (23.6)**	**23 (35.9)**	**86 (21.6)**	**0.028**
Echocardiographic right-left shunt after Valsalva				0.39
Mild	9 (1.9)	0	9 (2.3)	
Moderate	210 (45.5)	32 (50.0)	178 (44.7)	
Severe	243 (52.6)	32 (50.0)	211 (53.0)	
**Follow-up duration (years)**	**13.9 (13.0–15.3)**	**12.6 (13.5–14.2)**	**14.0 (13.0–15.4)**	**0.006**

Values are expressed as medians (interquartile range) or numbers (%), as appropriate. DVT, deep vein thrombosis; PE, pulmonary embolism; PFO, patent foramen ovale; TIA, transitory ischemic attack; The bold highlights those features with statistical difference.

**Table 2:** Procedural Characteristics.

	Overall	Elderly	Non-Elderly	*p*-Value
	(*n* = 462)	(*n* = 64)	(*n* = 398)	
Implanted device				0.31
Amplatzer	402 (87.0)	59 (92.2)	343 (86.2)	
Premere	23 (5.0)	1 (1.6)	22 (5.5)	
Atriasept	10 (2.2)	0	10 (2.5)	
Other	27 (5.8)	4 (6.3)	23 (5.8)	
Device size				0.52
≤20 mm	204 (44.2)	26 (40.6)	178 (44.7)	
21–25 mm	217 (47.0)	30 (46.9)	187 (47.0)	
≥26 mm	41 (8.9)	8 (12.5)	33 (8.3)	
Procedural time (minutes)	24.0 (19.0–33.0)	21.0 (16.8–29.3)	24.0 (19.0–33.0)	**0.022**
Fluoroscopy time	3.4 (2.4–5.0)	2.3 (3.2–4.3)	3.4 (2.4–5.0)	0.28
Radiation dose	411 (228–830)	354 (172–823)	416 (231–834)	0.29
Residual post-procedural R-L shunt	43 (9.3)	5 (7.8)	38 (9.5)	0.66
Mild	35 (7.6)	4 (6.2)	33 (8.2)	
Moderate	6 (1.3)	1 (1.6)	5 (1.3)	
Severe	0	0	0	
Antithrombotic therapy at discharge				0.57
Double antiplatelet therapy	459 (99.4)	63 (98.4)	396 (99.5)	
Anticoagulant with ASA or P2Y12i	3 (0.6)	1 (1.6)	2 (0.5)	

Values are expressed as medians (interquartile range) or numbers (%), as appropriate. ASA, acetylsalicylic acid; P2Y12i, P2Y12 inhibitors; bold highlights statistical differences.

## Data Availability

The data presented in this study are openly available in https://zenodo.org/uploads/11300858 (doi:10.5281/zenodo.11300858); accessed on 25 May 2024.
